# Extension for Prevention

**Published:** 1902-05

**Authors:** George S. Allan

**Affiliations:** New York


### EXTENSION FOR PREVENTION.[1]

BY DR. GEORGE S. ALLAN, NEW YORK.

In the excellent series of papers published in the *Dental Cosmos* for May, 1901, almost every phase and view of the theory of extension for prevention is ably presented. But little can be added except as it may present individual views *pro* or *con* on either side. I say this much with great reluctance, as I am prejudiced against hash in society meetings. Having committed myself, however, in a weak moment, and the chairman of your Executive Committee being obstinate and self-willed, I must make the best of it. The "dogma," or doctrine, however, is so radical in its nature and so contrary to ordinary conservative thought, that we may well afford to give it a little time and attention, even if we do get but little new meat in the effort.

We are accustomed to father the expression ex. for pre. on our able scholarly friend and brother Dr. Black, but Dr. Black in a late letter to me disclaims the honor, though he accepts the

[1] Read before The New York Institute of Stomatology, January 7, 1902.

greater one of being the real father of the principles of the dogma itself. Some one else "dubbed" it as the doctor expresses it. The first announcement of the doctrine was in a series of articles in the *Dental Cosmos* in 1891. Farther on in the letter I have referred to, Dr. Black writes: "You will see that all of my time is not spent in harping on ex. for pre., and also that I do not treat children precisely as I do grown-up folks, for reasons other than the conditions of the dentine." These two expressions taken together mean that Dr. Black's mind is broad enough to grasp the idea that no single dogma is great enough to cover the whole ground to the exclusion of others equally good and essential to correct practice. Most of you have undoubtedly read carefully the literature of the day on this subject, and come here with your judgment more or less decided as to its merits or demerits. Later on may be I will find out what they are. For myself I am convinced of two things. First, that with much that is good and valuable there is even more that is bad and dangerous in the dogma, ex. for pre., and that the dentist who gives it a ranking place in his daily work takes dangerous risks, and will probably regret it in the future. Secondly, the doctrine, except in name, is not new and in essentials is the same that Drs. Varney and Webb gave to the profession many years ago. Names, however, do not amount to much, and before a society like this may well be ignored.

If I were asked what the main business—duty—of the dentist was, I would say it was to save not only the teeth of his patients, but all of each tooth that was salvable, and in doing so I would add, to make certain that he gave a minimum amount of pain and discomfort joined to a maximum amount of success. The criticism, then, that I make on the dogma is, that it does not meet these requirements. The first thought that presents itself to my mind runs like this: Can it be wise and prudent to cut away sound tooth-substance in amount from two to ten times that actually required to remove the carious portion and properly shape the cavity in order to save the tooth? Is not such practice a sad confession of the inadequacy of the modern methods to meet conditions as we find them? And further, is it not well to pause before adopting it, to consider well this fact,—viz., that no amount of care or skill can ever replace what you have so recklessly cut away in case the lapse of time proves that you have made a mistake, and that you have not put in a filling that will last "a hundred years"

or "forever." And who can say that an immune period of decay may not recur, and thus do away with the necessity of extensive cutting? Such an uncertainty might well call a halt to the most enthusiastic. One must, indeed, know well what a master of technic he is and how great his knowledge and skill to take such risks, and to be able to say to his patients I know what I am about, and you must accept my doctrine unquestioned. There is no other road to success. This one does not admit of failure.

The dogma has as a foundation certain well-recognized facts pertaining to the origin and early stages of caries of the teeth. The existence of these conditions was recognized and made the basis of all active procedures for the prevention and arrestation of caries long before the reason why was known or admitted of scientific explanation. Now that it is known, and for nearly all practical purposes, every phase and form of the destructive work comprehended we are enabled to work, not so much more surely as to results, but far more intelligently. Many things in this world have been done well "by the rule of thumb," as the expression is, that in the end have been done better where this same rule is well lighted by knowledge; and, what is of more importance, more improved methods have taken the place of the more crude ones based on experience and personal skill only, and a larger number of good workers has followed as a necessary sequence. From a very early date it has been known that caries always worked from without inward,—from the surface of the tooth towards the centre,—and those intelligent, skilful men who excited the admiration of the profession in its infancy had a reason for the faith that was in them, and knew well what they were about when they said in effect that cleanliness was the first law in the salvation of the teeth, and a perfect stopping the next. It covers the ground to-day just as perfectly as it did fifty years ago, when Dunning, Maynard, Townsend, and others led the way. But this is a bit of a digression.

W. D. Miller first gave a rational explanation of the causes that make caries of the teeth. Too much credit cannot be given him for his long laborious work ending in such complete success. No one questions to-day that the baleful germs he brought to light are not only the first cause of the disease, but the continuing and ending one as well. In one respect only did he fail to change the views previously held,—viz., that the decay commenced on the outside of the tooth, that the germs in growing as a waste product secreted an acid, and that this acid chemically destroys the teeth briefly states the case. The new doctrine takes hold of Miller's work, and, by the use of so-called gelatinous plaques (see *Dental Cosmos,* 1897, articles of Dr. J. L. Williams), shows that there are certain areas of the tooth more liable to decay than others,[1] and that the durable filling must not only take the place of the carious portion of the tooth, but that a much more extended portion, which, though not affected, lies in the zone of liability, will, if left alone, probably decay, from the fact of its not being enclosed in a self-cleansing arc. Where decay is rapid in growing mouths, or, for that matter, at any period in life, gold as a filling-material is contraindicated. It is better and safer practice to resort to either gutta-percha or amalgam to tide over these years. Better results are sure to follow. Again and again have I seen practically complete arrestation of decay follow these methods, and been enabled at a later date to permanently safe-guard the teeth with gold.

Two thoughts have naturally presented themselves. First, can this zone of liability be accurately mapped out, so that it will cover neither more nor less than enough of tooth-substance to meet the requirements. To read the statements of the advocates of the new method, one would suppose the problem was an easy one to solve. No great importance seems to be attached to it. It is taken for granted that a little thought and study, following definite well-laid-out lines, is all-sufficient, but I for one do not agree with this opinion. Approximately it may be in some cases, but my belief is that from the very nature of things it is an indeterminate problem, one in which the guiding factors are ever changing as environment, personal habits, or conditions in life change, and cannot be made to conform to fixed laws and directions, and in no two cases can similarity in all the factors be predicated. This of itself would be enough to make the cautious man hesitate and doubt, not only his own judgment, but the judgment of the sponsors of the dogma. The true professional instinct might step in and say, I cannot make this sacrifice of sound tooth-substance, where there is so much uncertainty; and, if they see clearly they might add, My conscience will not permit me to do this thing; and if they have carefully read

---

[1] This was known before, but the real meaning was not known,—viz., that the plaques hold the germs against the surface of the tooth.

the able, clean-cut paper of Dr. Safford Perry, they might also add, My reverence for the natural teeth is too great to permit of it.

Secondly, accepting the fact that there is a zone of liability to decay more or less clearly defined; that certain portions of the contiguous territory of the carious teeth are more vulnerable than others, the all-important question to ask is, cannot these danger points be as well safe-guarded in some other and more conservative way? For an answer to this question let me read to you from a little but very valuable book, the title to which is "Lectures on Operative Dentistry," kindly sent me by the author, Dr. G. V. Black, in answer to some inquiries I made by mail. My answer to this then is, a little better and safer method is at hand, and the putative author of the dogma ex. for pre. puts it in a nut-shell for us. What is true of the buccal surfaces is equally true of all other tooth-surfaces. The law holds good in all mouths and under all conditions, and we have but to follow where it leads to insure success. I say, then, make your fillings in the minor cavities equally good with those in the major ones, and protect the area of liability by strict attention to the laws of prevention of decay by enforcing cleanliness on the part of the patient, and see that you do your own part faithfully and at regular intervals. It is a curious coincidence of dental thought that at the very time that ex. for pre. is being so vigorously pushed and advocated, that other one of insuring the salvation of the tooth by a rigid adoption of the means of keeping them clean, both on the part of the patient and of the dentist, should also be most strenuously pushed. I am glad it is so, for it will help many doubtless to a more careful consideration of the whole question.

Allow me to present some other views of the good and bad points of the radical and conservative methods of practice. Maybe they will do good. The claim made is, that by sacrificing in the beginning that portion of the healthy tooth adjacent to the cavity, which from its position is liable to decay, and including it in the cavity, thus extending the walls of the cavity to areas immune to decay, permanent results are obtained, and the face of the tooth so treated and well filled will not decay again. The operation becomes a permanent one in every sense of the term. What more could one ask for? I omitted to say that the advocates of this principle maintain that such operations can be both successfully and rapidly made, and without giving an undue amount of pain or discomfort. Of course, the contour and finish will be in the most approved manner. In the old days of Varney and Webb such cutting away of tooth-substance was accompanied by great pain and suffering, of which I have personally the most feeling recollections.

I do not understand how "three or four cuts with a large Wedelstaedt obtuse hoe will so extend the cavity at the gingival margin that when the filling is completed it will be covered with a healthy gum septum," but I accept what Dr. Wedelstaedt says as true in his hands. Times change, indeed, but I doubt much whether human pain and suffering have changed as radically, even when the modern improved tools are in the hands of the most expert. But little if anything is said on another point, to which I will now draw your attention.

We all know how approximately large gold fillings to the region of the pulp invites sensitiveness and irritation, and oftentimes inflammation and the subsequent death of the pulp. Why this neglect? The danger is as real to-day as it ever was. To be sure the claim is made that the work is rapidly done, but the effect in the end must be the same. Dr. Black says (*Dental Cosmos,* July, 1891, page 537), "If the tooth survives the operation, as unquestionably the most of them do, and sensitiveness to thermal changes ceases, still other pathological changes may result. The irritation ofttimes invites the formation of secondary dentine in the pulp, especially in that portion near the floor of the cavity, and the vitality of the tooth as a whole is lessened." And there is no doubt that liability to cleaving and breaking is greatly increased. It is well here to notice the claim made for rapid work. The old school recognized certain physical properties in gold, which could not be altered or amended. The relation between force and metal was a constant one, and did not permit of change. To pack gold thoroughly,—and no one believes more decidedly in this real packing of gold and producing a solid filling than Dr. Black,—the force required must be in direct proportion to the size of the pellet, pad, or cylinder to be condensed, welded into a homogeneous metalic mass. Now, the rapid work of necessity means the use of large pellets, and these in turn mean much greater force and care in packing, or a softer, less compact filling. There is no getting away from this statement of law and facts. Varney and Webb,

Maynard and Dunning, all advocated small pellets, small points, and accepted slow progress as an unavoidable necessity.

Lastly, the objection to the large and unsightly gold fillings is a real one, and rests on a solid foundation. I am thankful that the demand for art that does not disfigure and make ashamed is on the increase. More and more do I find patients demanding the hiding or removal of the yellow, shining golden signs from their mouths and the substitution of something more in harmony with nature's perfect work. As one patient put it, "I want to smile without twisting the corners of my mouth out of shape."

Dr. Johnson's book is one of the very best on operative dentistry that has been given to the profession, and ought to be in the hands of all for study and ready reference, and yet see what a strange practice he advocates. On page 83 he says, "Teeth breaking down in early life where caries is progressing rapidly require the most strenuous means to bring them under control, and this means with him the most heroic cutting in order to remove not only that portion which has decayed, but that other and much more extended portion which may decay, so that safety and permanence may be assured." At this early age—the term is a little ambiguous and indefinite, to be sure, but probably includes the period from twelve to sixteen—at least the roots of the teeth are not fully formed. The pulp is full of life, vascular and sensitive to a great degree, and relatively fills its maximum portion of the interior of the teeth, and yet in these cases he would give the dogma full swing, carry it to its fullest extent. To such an audience as this it is needless for me to say what that means.

To my mind the really modern, up-to-date practice draws the line sharply between the heroic method and the conservative one, and in all doubtful cases adopts the latter, and children and all others where caries is rapid and progressive are classed in the conservative list. Moreover, they look upon this latest Western fad as being new only in name. The methods of shaping cavities are modifications only of the old, adapted to Dr. Black's theories regarding the treatment of the enamel margins. The ways of packing the gold, the shaping, contouring, and finishing the margins, especially the gingival ones, are matters of history, excepting again the extension of the gingival margins, and I question very much if there will be found on examination any great fundamental difference in the instruments used or in the method of handling them; and what is of more importance, the limitations of its usefulness and its exact position as a means of saving teeth are more clearly defined. Experience is a rough teacher, but he does his work well, and all have to acknowledge his edicts.

It is amusing to read the cases reported by the new school as proofs of their position. Poor, sloppy, careless work has many sins to answer for, but no one of them is greater than this one of being made the father of ex. for pre.'s great merits. As I read the reports, most of the failures reported are attributable to the dentist's lack of care or ability, and indicate the probability that in the same hands ex. for pre. practice would show even worse results.

The only reason why the methods advocated by Drs. Webb and Varney were not adopted generally, confined though they were to special cases, was on account of the inherent difficulties met with in putting them into practice, coupled with the pathological sequences, frequently resulting in invalidating and worse than invalidating the otherwise most successful operation.

It is a mistake to say that the Varney and Webb methods were not accepted by their contemporaries. In truth, they were accepted, not only then, but they hold their own to this day. They were and are rejected only by those few in number who could not or cannot attain unto them, for many and divers reasons, principally those related to incapacity or ignorance.

The quotations given by Dr. Wedelstaedt, taken from the "American Text-Book of Operative Dentistry," in support of this statement are wholly irrelevant and lacking in force. The quotations are taken from chapters relating to composition fillings,—fillings composed of two or more different materials,—and in no way directly or indirectly refer to the Varney methods, and describe only the proper modes of procedure where such ways are indicated. Granting, though, that the Varney-Webb methods were rejected,—which is a mistake,—it in no way disproves the position taken by so many that the principle and practice so loudly proclaimed and strenuously advocated as new and peculiar is neither one nor the other, but is old, and in this time of rapidly moving events and changes might even be said to be venerable with age. It has been weighed in the balance and found wanting, but only in those qualities which contraindicate its adoption, except in special cases. Where it is good it has been found to be very good, and where it is bad it has been found to be bad indeed.

In closing allow me to again quote Dr. Black's remarks, as found on page 142, "Lectures on Operative Dentistry:"

"It sounds the key-note of the successful finished practice of to-day, a practice that, backed by the knowledge of causes and effects so recently acquired, enables us to save practically all that is salvable of the infected tooth or teeth of our patients, and its advocates thoroughly believe that it is better to trust to thoughtful, intelligent, persistent care and attention to keep the teeth clean and safe, where indications are bad through the period of danger, than to Providence backed by a mutilated tooth and a disfigured face."

On page 830, *Dental Cosmos,* September, 1899, Dr. Black says, "Why not, then, make an honest attempt to tide over this dangerous period by cautious consideration in shades rather than resort to the destructive ones?"

It is well, though, and wise to inquire carefully into the causes that have brought this so-called new departure so prominently into notice. There must be a reason, and a good one, to make such men as are back of it—men so earnest, honest, and capable, commanding our respect and confidence to so great an extent—take the position they do. Real evils must be in existence that they wish to remove, real dangers to avoid, or they would not be found fighting so grandly for the faith that is in them. I honor them for it. I like them exceedingly, and am only sorry I do not know them all personally. Those who do not accept in full measure all that is said and written in advocacy of ex. for pre. must think, act, and work also. They have much to do, especially in the way of explaining, and they will also have some things to apologize for that may not contribute to their peace of mind.

I take it that the real strength and force of the new movement will be found to lie just here. Too much poor work has been done, too many salvable teeth are being lost, and in general the profession does not live up to its standards, or equal the expectations placed in it. Ex. for pre. is a protest against the imperfect, neglectful services of the day. It takes its life from the belief that the average man cannot command good work at all times, or that continuing care and attention that means safety and comfort. Therefore the one complete operation must be made to bear the burdens of the future, as well as the necessities of the present. Maybe too much reliance is being placed on this single operation, and better results can be obtained by other means, extended though they may be through longer periods of time.

It would be difficult to define clearly what the best modern practice is. Conditions, details, and methods vary so much, but the fundamental controlling thought may be expressed in the diction, a clean tooth-surface will not decay. So all the energies of the operator are directed to obtain and maintain this condition. As eternal vigilance is the price of liberty, so also is it the one perfect way to the salvation of the teeth.

If the carious portion of a tooth be carefully removed and the cavity thus formed be kept clean and free from caries producing germs, no fresh decay will follow. The impossibility of doing this thing makes the filling necessary, so that a continuous smooth surface, which can be kept clean and free from germs, may be obtained. In no case is there any saving virtue in the filling-material itself, unless it may be in some of the copper amalgams. At the best a filling-material is a necessity which ought and must be limited in amount to replacing the lost portions of the tooth only. Any excess of filling-material beyond these requirements means the weakening and impairing the remaining portions of the tooth.

As the preparing and shaping of the cavity is primarily to remove completely the carious portions, too much care and attention cannot be given to it; but almost equal pains must be given to that other necessity, the so shaping the cavity that a perfect filling may be inserted—one so perfect that no moisture can find access to the walls of the cavity. For this purpose, and this only, some portions of the walls of the cavity may have to be cut away. If gold is the filling-material employed, the access to the cavity must be so enlarged that direct pressure may be directed to all portions of the cavity in order to obtain perfect condensation and adaptation. Varying skill and technic on the part of different operators, of course, make great differences as to the amount and kind of removal of tooth-substance. What I want to get at and give emphasis to is that it takes more skill and ability to fill the small approximal cavities, from one to two millimetres in diameter, especially between molars and bicuspids, and make perfect work, than it does the larger ones, the access to which is so much easier. Given, though, the perfect filling, let it be large or small, and keep the surfaces clean by faithful attention on the part of both dentist and patient, and tooth salvation results, with many advantages accruing in favor of the small filling. Small fillings in the approximal surfaces of molars and bicuspids fail to fulfil their mission in greater degree than the larger ones, only because they, as a rule, are more imperfectly made, and for no other reason.

